# Survival Outcome of Surgical Resection vs. Radiotherapy in Brain Metastasis From Colorectal Cancer: A Meta-Analysis

**DOI:** 10.3389/fmed.2022.768896

**Published:** 2022-03-08

**Authors:** Yu Chang, Chia-En Wong, Po-Hsuan Lee, Chi-Chen Huang, Jung-Shun Lee

**Affiliations:** ^1^Section of Neurosurgery, Department of Surgery, College of Medicine, National Cheng Kung University Hospital, National Cheng Kung University, Tainan, Taiwan; ^2^College of Medicine, Institute of Basic Medical Sciences, National Cheng Kung University, Tainan, Taiwan; ^3^Department of Cell Biology and Anatomy, College of Medicine, National Cheng Kung University, Tainan, Taiwan

**Keywords:** metastasectomy, radiotherapy, surgical resection, colorectal cancer, brain metastases

## Abstract

**Background:**

The incidence of brain metastasis from colorectal cancer (CRC) increases along with the greater survival rate for CRC because of the advances in therapeutic modalities. Local treatment strategies for brain metastasis include surgical resection and radiotherapy. Nevertheless, given the incongruent literature, the optimal therapeutic approach remains to be investigated. This study aims to systematically compare the real-world survival outcome of surgical resection and radiotherapy in patients with brain metastasis from CRC.

**Methods:**

Following Preferred Reporting Items for Systematic Reviews and Meta-Analyses (PRISMA) and Meta-analysis Of Observational Studies in Epidemiology (MOOSE) guidelines (PROSPERO, ID: CRD42021240200), the Cochrane Library, Embase, and Medline were searched from the inception of the database to August 2021. Meta-analyses were conducted with results pooled using hazard ratios with corresponding 95% CIs to evaluate the overall survival (OS) following local treatment for brain metastasis from CRC. Summary effects were evaluated using a series of random-effect models.

**Results:**

In this review, 17 retrospective studies comprising 1,438 participants were included. In comparison with radiotherapy, the OS of patients who received brain metastasectomy was generally longer (HR, 0.53; 95% CI, 0.47–0.60). Extracerebral metastases (HR, 1.58; 95% CI, 1.34–1.86) and multiple brain metastases (HR, 1.38; 95% CI, 1.10–1.72) were associated with worse survival outcomes.

**Conclusions:**

For patients with brain metastasis from CRC, the current real-world evidence demonstrated the survival benefit of aggressive neurosurgical management in suitable patients. Additionally, patients with extracerebral metastases and multiple brain metastases had worse survival outcomes.

**Systematic Review Registration:**

https://www.crd.york.ac.uk/PROSPERO/display_record.php?RecordID=240200.

## Introduction

With advances in therapeutic modalities, the survival rate in patients with colorectal cancer (CRC) is greater than before (1). Since the blood–brain barrier impedes the penetration of most chemotherapeutic and biologic agents, the development of brain metastases (BM) increases along with survival, wherein up to 13% of patients with CRC have various forms of BM (1, 2). Regarding the time interval from primary CRC to BM, most BMs are found to be metachronous, whereas other BMs are synchronous (3, 4). Supratentorial BM is more frequent than that in the posterior fossa, and solitary metastases account for 40–60 % of the cases (5–7). Most patients have poor outcomes despite aggressive treatments, including surgery, radiotherapy, radiosurgery, and systemic therapy. The reported median survival time (MST) after the diagnosis of BM varies from 2.5 to 87 months in the literature (6, 7).

Concomitant extracerebral metastases (ECM) develop in most cases of BM, predominantly in the lungs and liver (8). Aggressive surgical metastasectomy of the hepatic and pulmonary metastases from CRC is a standard treatment strategy with benefits to survival outcome (9, 10). However, the efficacy of surgical resection of BM remains uncertain in patients with primary CRC. Although some studies have reported a longer overall survival (OS) in patients who underwent neurosurgical resection than in those receiving whole-brain radiation (WBRT) (11–13), discrepancies were found in the results of observational studies (14, 15). Moreover, the impact of newly developed multimodal BM-directed therapeutic approaches, such as stereotaxic radiosurgery (RS) and combined surgery with RS, was addressed in only a few studies (16). Although some retrospective studies aimed to identify outcome predictors and optimize treatment strategies, the relatively low incidence and the paucity of available comparisons between treatment modalities hindered the establishment of a treatment consensus for colorectal cancer with brain metastasis (CRC BM), which remains in a case-by-case fashion currently.

Given the incongruent literature, the optimal therapeutic approach for CRC patients with BM remains to be investigated.

To assess the efficacy of treatment strategies for CRC BM in the literature, including surgical resection, or radiotherapy, we performed a comprehensive review, as well as meta-analysis, of published literature.

## Methods

The present systematic review and meta-analysis were based on the Cochrane Handbook for Systematic Reviews and Interventions (17). The results were reported in accordance with the Preferred Reporting Items for Systematic Reviews and Meta-Analyses (PRISMA) and Meta-analysis Of Observational Studies in Epidemiology (MOOSE) guidelines (Supplementary Methods 1, 2). This study was registered on the online platform PROSPERO (ID: CRD42021240200). The Cochrane Library (United Kingdom), Embase (Netherlands), Medline (United States) electronic databases were searched from the inception of the database until August 2021. Two independent investigators (YC and CEW) executed the search to identify relevant studies for inclusion. Discrepancies were resolved by a senior reviewer consultant (JSL) or by consensus. The exhibiting details of the search are presented in Supplementary Method 3.

### Eligibility Criteria

The English-language articles with the following criteria were included: (1) randomized controlled trials and prospective or retrospective cohort studies, except conference abstracts, letters to the editor, case reports, editorials, and review articles; (2) the studies of adults with CRC BM; (3) the studies reporting comparative survival outcome of surgical resection vs. radiotherapy and using OS as an endpoint. Studies with insufficient data to calculate the hazard ratio (HR) to estimate the treatment effect were excluded. In cases of duplicate studies with an accumulating number of patients or different follow-up periods, only the one with the longest follow-up duration was included.

### Data Extraction

Two investigators (YC and CEW) independently extracted the following data from eligible studies: first author's last name, year of publication, characteristics of the patients, and treatment strategies for patients with BM from CRC.

### Quality Assessment

Two investigators (YC and CEW) independently completed a critical appraisal of the included literature using the Risk of Bias In Non-randomized Studies of Interventions (ROBINS-I) (18) tools. Additionally, a senior reviewer (JSL) addressed any item on which assessors did not reach the consensus.

### Statistical Analysis

Statistical analyses were performed using the functions available in the metafor package (19) within the R Studio software, United States, (Method 4). Survival outcomes after BM were obtained by extracting the HR directly from each reference. When studies did not report the HR but was rather presented as the Kaplan–Meier survival curves, the estimated HR from these curves were obtained through a well-established method (20) using a calculation spreadsheet developed by Tierney et al. (21).

Hazard ratios (HRs) from the included studies were pooled through the inverse variance method using the random-effects model with the DerSimonian and Laird method (22) adopted for heterogeneity estimation. Furthermore, we aimed to alleviate the statistical and conceptual heterogeneity of our meta-analysis by performing subgroup analysis. For studies reporting multivariate risk factors or prognostic factors for OS, we also acquired HRs and pooled them in the meta-analysis.

The effect sizes were presented with their corresponding 95% CIs. Heterogeneity was assessed using *I*^2^ statistics proposed by Higgins and Thompson (23, 24), with *I*^2^ < 25%, 25% < *I*^2^ < 50%, and *I*^2^ > 50%, thereby indicating low, moderate, and high heterogeneity, respectively.

### Publication Bias

For meta-analyses including more than 10 studies, we used a funnel plot to detect publication bias. The Egger's test was performed to indicate significant asymmetrical distribution with *P* < 0.05.

## Results

### Study Selection

**Figure 1:**
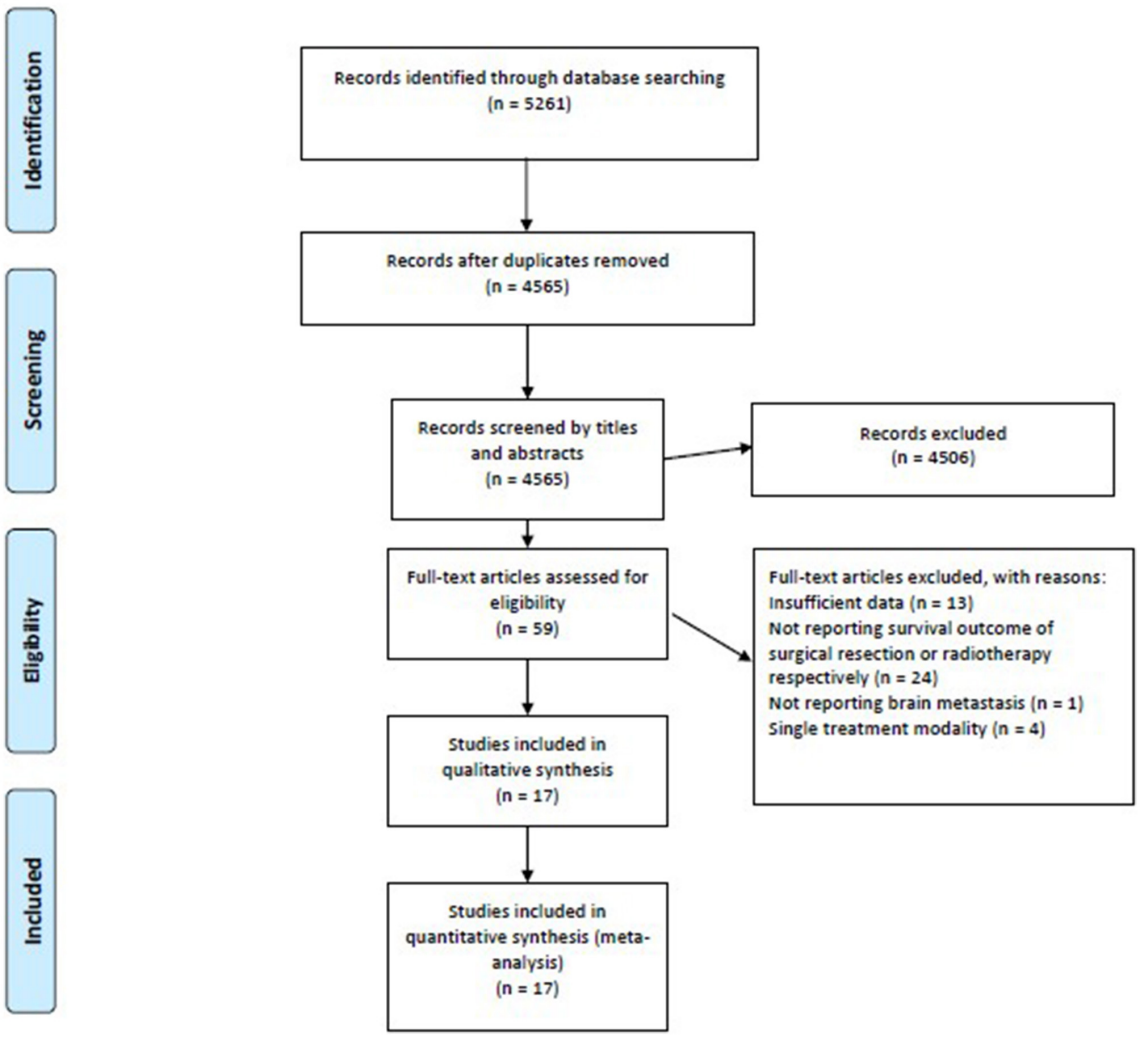
The PRISMA flow diagram demonstrates a total of 5,261 potential references were extracted initially, and the meta-analysis included 17 studies meeting the eligibility criteria. PRISMA, Preferred Reporting Items for Systematic Reviews and Meta-Analyses.

Our search strategy identified 5,261 references from the Cochrane Library, Embase, and Medline electronic databases. After screening the titles and abstracts, we excluded duplicates (*n* = 696) and irrelevant references (*n* = 4,565). The remaining 59 studies were retrieved for a full-text review, 17 of which were considered eligible for qualitative and quantitative syntheses (Figure 1).

Supplementary Table 1 summarizes the excluded articles after a full-text review.

### Study Characteristics

As shown in Table 1, a total of 17 retrospective cohort studies (4, 8, 11–13, 15, 25–35), involving 1,438 patients with CRC BM were included. Among the 17 studies, 11 (4, 8, 12, 13, 25–27, 29, 30, 32, 34) studies clearly pointed out the radiation modalities with either WBRT or RS, whereas the remaining 6 (11, 15, 28, 31, 33, 35) did not.

**Table 1:** Characteristics of included studies.

**Study**	**No. of patients (male %)**	**Age, median (range)**	**Interval from CRC to BM, months, median (range)**	**KPS ≥70, *n* (%)**	**RSC, *n* (%)**	**ECM %, Li/Lu/none**	**Single BM, *n* (%)**	**BM supratentorial limited, *n* (%)**	**Treatment, *n* (%)**	**MST after BM by treatment, months**	**WBRT dose**	**RS dose**	**CT, *n* (%) before/after BM**
Alden et al. (25)	19 (NA)	66 (43–87)	32.1 (0–100)	N/A	N/A	53/68/21	12(63)	10 (53)	WBRT: 14 (73.7) Sx: 5 (26.3)	WBRT:4.9 Sx :2.6			
Hammoud et al. (26)	100 (62)	61 (31–90)	26 (NA)	60 (60)	65 (65)	52/71/5	64 (64)	48 (48)	WBRT: 57 (57) Sx±WBRT: 36 (36)	WBRT: 3 Sx±WBRT: 9	3,000 cGy/10		
Baek et al. (4)	118 (53)	54 (19–77)	12.2 (0–76.2)	64 (54)	89 (75)	45/75/NA	58 (50)	55 (48)	Sx ±WBRRT: 24(20) RS: 8 (7) WBRT: 74 (63) Local RT: 12 (10)	Sx ±WBRRT: 7.2 RS: 6.1 WBRT: 4.9 Local RT: 3	3,000 cGy/10	1,900 cGy (median)	NA/34 (29)
Jung et al. (27)	126 (62)	64 (30-81)	28.7 (0–139)	42 (33.3)	95 (75)	32/72/9	50 (39.7)	61 (48)	Sx ±WBRRT: 20 (16) RS: 41(32) WBRT: 45 (36) S: 20 (16)	Sx ±WBRRT: 11.5 RS: 9.5 WBRT: 4 S: 1.5	3,000 cGy/10		67 (53)/41 (33)
Fokas et al. (28)	78 (39)	NA	20 (0–84)	39 (50)	NA	NA/NA/36	NA	NA	Sx: 19 (24) RT: 59 (76)	Sx: 10 RT: 3.6	3,000 cGy/10	2,000 cGy (median)	49 (63)/NA
Damiens et al. (8)	48 (52)	63 (37-84)	NA	NA	29 (60)	50/64/10	30 (63)	26 (54)	Sx: 2 (4) Sx + WBRT: 16 (33) WBRT: 22 (46) S: 8 (17)	Sx: 3 Sx + WBRT: 13 WBRT: 4 S: 2			
Kye et al. (29)	39 (59)	59 (40–81).	32.3 (0.5–76).	NA	22 (56)	41/80/NA	24 (62)	23 (59)	Sx :6 (15) RS: 9 (23) WBRT: 20 (51) S: 4 (11)	Sx :15.2 RS: 6 WBRT: 4.4 S: 2			
Noura et al. (15)	29 (79)	61 (48–74)	34.3	NA	17 (59)	26/74/22	9 (31)	14 (48)	Sx ± RT: 17 (58) RT: 8 (28) S: 4 (14)	Sx ± RT: 9.2 RT: 8.7 S: 2.8			
Kim et al. (30)	38 (66)	63 (52-69)	NA	37 (97)	22 (60)	NA	21(55)	26 (68)	Sx ± WBRRT: 11 (29) RS ± WBRRT: 27(71)	Sx: 16.2 RS: 5.6	3,000 cGy/10		31 (82)/19 (50)
Magni et al. (11)	41 (61)	58 (23-75)	36 (0–116)	NA	24 (58.5)	37/88/5	22 (54)	24 (59)	Sx± CT ± RT: 12 (30) RT + CT: 9 (22) RT: 12 (30) S: 6 (15)	Sx ± CT ±RT: 21.4 RT + CT: 11.9 RT: 4.2 S: 2.1			18 (45)/17 (41)
Suzuki et al. (12)	113 (65)	NA	22.8 (0–128)	NA	49 (43)	37/66/22	NA	56 (50)	Sx + WBRT: 63 (56) RS: 9 (8) WBRT: 30 (27) S: 11 (10)	Sx + WBRT: 10.5 RS: 5.1 WBRT: 3.1 S: 1.2	5400 cGy/5.6 weeks		
Fountzilas et al. (31)	40 (68)	56 (34–78)	NA	36 (90)	20 (50.0)	48/75/10	14 (35)	17 (43)	Sx + RT: 9 (23) RT: 26 (65)	Sx + RT: 21.4 RT: 2.9			30 (75)/9 (24)
Tapia Rico et al. (32)	59 (49)	65 (38–87)	24 (4–72)	NA	20 (34)	47/54//NA	NA	NA	Sx + WBRT: 27 (46) WBRT: 27 (46)	Sx + WBRT: 8.5 WBRT: 2.2			
Del Carpio Huerta et al. (30)	28 (64)	64 (21–81)	36 (NA)	24 (85)	NA	35/71/14	19 (76)	NA	Sx ± WBRT: 14 (50) WBRT: 12 (43)	Sx ± WBRT: 12.1 WBRT: 4.6			NA/10 (36)
Lu et al. (33)	80 (65)	58.4 (NA)	NA	40 (50)	50 (63)	NA/70/16	44 (55)	53 (66)	Sx+CT: 15 (19) Sx: 4 (5) RT + CT: 27 (34) RT: 16 (20) CT: 18 (23)	Combined Tx:11 Single Tx: 4	3,000 cGy/10	1,200 to 2,200 cGy	NA/60 (75)
Boysen et al. (34)	235 (50)	64.8 (NA)	NA	NA	115 (49)	NA	NA	NA	Sx: 158 (68) RS: 51 (21) Sx+RS: 26 (11)	Overall: 9.6			
Bonadio et al. (35)	247 (50)	62.9 (22–93)	27.6 (0–141).	NA	NA	NA/NA/6	128 (52)	NA	Sx: 43 (17)t Sx + RT: 58 (24) RT: 76 (31) S: 70 (28)	Sx: 2.5 Sx + RT: 7.0 RT: 2.5 S: 0.6			

*BM, brain metastasis; cGY, centigray; CRC, colorectal cancer; CT, chemotherapy; ECM, extracranial metastases; Li, liver; Lu, lung; MST, median survival time; KPS, Karnofsky Performance Score; RS, radiosurgery; RSC, rectosigmoid cancer; RT, radiotherapy; S, supportive treatment; Sx, surgical resection; Tx, treatment; WBRT, whole-brain radiotherapy*.

### Quality Assessment of Included Studies

Supplementary Table 2 demonstrates the summary of ROBINS-I of the included studies. No critical risk of bias was detected. Ten studies were assessed as serious risk of bias, and seven studies as the moderate risk of bias.

### Survival Outcome

**Figure 2:**
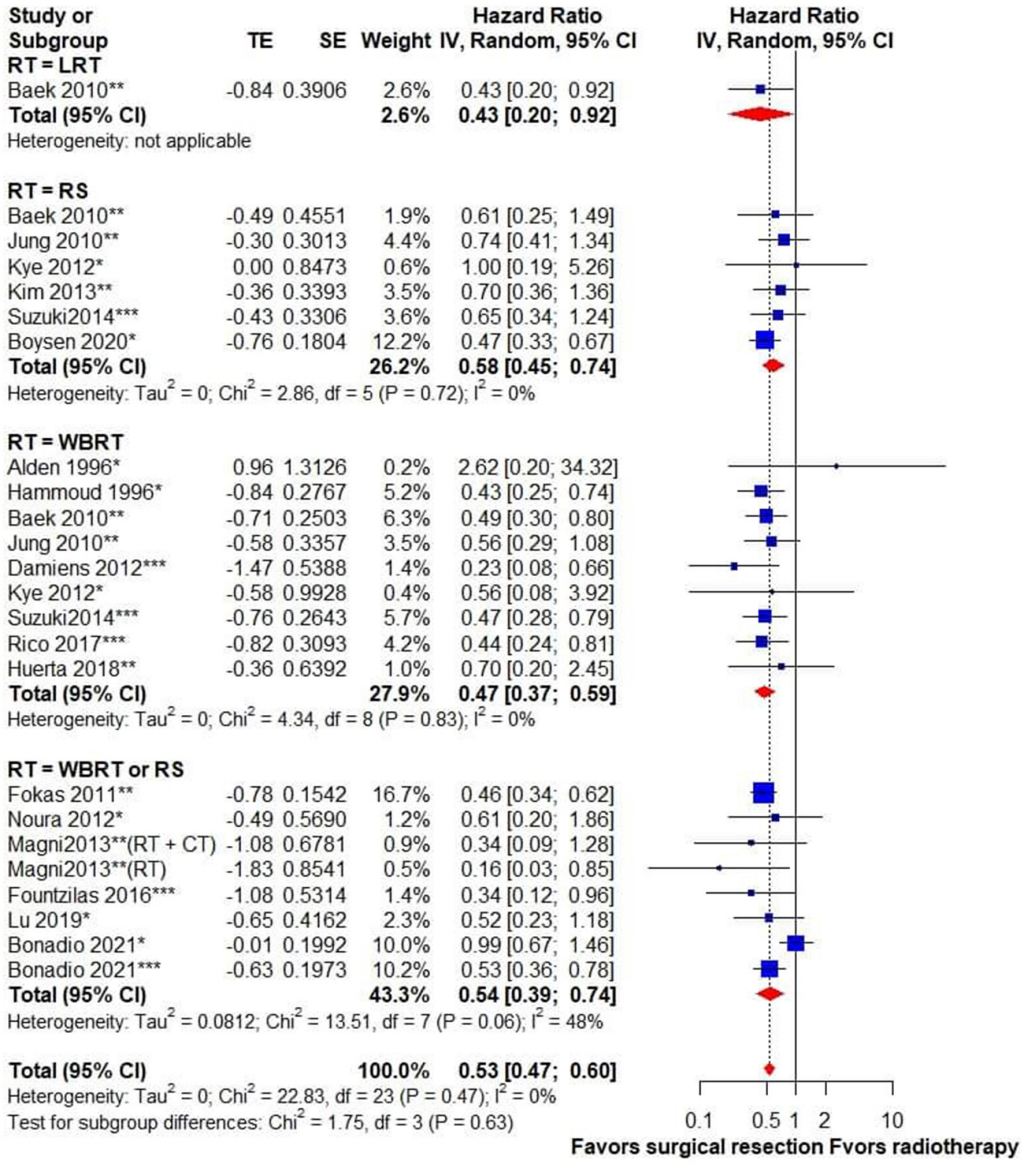
Forest plot for overall survival in patients receiving surgical resection vs. radiotherapy for brain metastasis from colorectal cancer. Pooled HR with 95% CI was calculated under random-effects models. HR, hazard ratio; CI, confidence interval; CT, chemotherapy; LRT, local radiotherapy; RT, radiotherapy; RS, radiosurgery; WBRT, whole-brain radiotherapy. ^*^Surgical resection alone, ^**^surgical resection ± RT, ^***^surgical resection + RT.

Regarding the survival outcome of treatment modalities for CRC BM, we compared the OS of patients receiving metastasectomy with those treated with radiotherapy. The surgical group exhibited better survival outcomes than the radiotherapy group, and no statistical heterogeneity was detected (HR, 0.53; 95% CI, 0.47–0.6; *I*^2^ = 0%) (Figure 2).

### Subgroup Analysis

To identify the impact of different radiation modalities on the survival outcome, a subgroup analysis of surgery vs. WBRT and RS was performed. It indicated that surgery was associated with superior survival outcome when compared with either WBRT (HR, 0.47; 95% CI, 0.37–0.59; *I*^2^ = 0%) or RS (HR, 0.58; 95% CI, 0.45–0.74; *I*^2^ = 0%). Subgroup analysis based on univariate or adjusted HRs showed no statistical subgroup differences (Supplementary Figure 1). We also performed a subgroup analysis by classifying studies published within 5 years (after 2016) or more than 5 years ago (before 2016), and there was no subgroup difference (Supplementary Figure 2). Subgroup analysis based on different sample sizes showed similar results in studies with different case numbers (Supplementary Figure 3).

### Prognostic Factors

The pooled result of prognostic factors from the included studies indicated that patients with ECM (HR, 1.58; 95% CI, 1.34–1.86; *I*^2^ = 10%) and multiple BM (HR, 1.38; 95% CI, 1.1–1.72; *I*^2^ = 56%) had worse survival outcome (Supplementary Figures 4, 5). Whether BM developed metachronously or synchronously was not associated with the survival outcome (HR, 2.05; 95% CI, 0.89–4.77; *I*^2^ = 0%) and the primary CRC location (rectum or colon) had little impact on the OS (HR, 0.8; 95% CI, 0.35–1.82; *I*^2^ = 89%) (Supplementary Figures 6, 7).

### Publication Bias

The visually symmetrical funnel plot with Egger's test result (*p* = 0.85) indicated no potential publication bias (Supplementary Figures 8, 9).

## Discussion

### Key Findings

This is the first meta-analysis to compare different treatment modalities for CRC BM in terms of survival outcome. Our findings suggested that patients undergoing surgical resection of BM were associated with longer survival. Moreover, these findings would be reinforced by the large sample size and low heterogeneity across the included studies.

### Surgical Resection vs. Radiotherapy

Brain metastasis was considered an end-stage disease because of its poor survival. Given the inevitable craniotomy-related surgical risks and the advance in radiotherapeutic techniques, a large group of patients with CRC BM opts for non-surgical radiation modalities as local treatment.

Neurological improvement after WBRT has been reported in BM from various types of cancer (36). However, the majority of reported MST after WBRT for CRC BM was <5 months (3, 4, 6, 12, 16, 25, 26, 28, 32). In contrast, the MST after BM has been reported to be over 10 months in patients treated surgically (6, 7, 12, 13, 28). Since metastatic CRC harbors a unique survival benefit from aggressive metastasectomy in hepatic and pulmonary metastases, we drew attention to the potential benefit of brain metastasectomy in CRC. The result of the pooled analyses provides quantitative evidence indicating that patients who received surgical resection of BM were associated with longer survival compared with those receiving WBRT.

The recent advances in radiosurgical modalities in recent years including Gamma Knife or CyberKnife, which can precisely deliver high-dose radiation to lesions <2.5 cm, can be applied to single as well as multiple lesions simultaneously (37, 38). Therefore, RS has drawn attention in the treatment of CRC BM and was proposed as either an alternative or an add-on to WBRT (4, 27, 30). However, incongruent results were reported. While Matsunaga et al. (39). have reported the benefit of RS for the suppression of local tumor growth in their single-arm study, the survival time in patients treated with RS was not longer than those receiving metastasectomy in other studies (12, 30, 34). Through a more robust statistical method, our analysis indicated that patients undergoing surgical resection of BM exhibited better survival outcomes than RS.

The current surgical indications of elective BM resection, including stable systemic disease, Karnofsky performance score (KPS) of >70, and surgically accessible lesion (40), were largely based on the previous observations in patients with primary lung cancer, breast cancer, and renal cell carcinoma. These indications may not reflect the real applicability in CRC BM, and more patients could potentially benefit from surgical resection. Further investigation using well-designed prospective studies may provide clearer indications for surgical resection of CRC BM.

Although the survival benefit of BM metastasectomy was observed in our analysis, this beneficial effect may be potentially associated with the adjuvant radiotherapy administered following surgical resection. The surgical group contained patients receiving radiotherapy in combination with surgery in several studies (8, 11, 27, 28, 30, 32). Recurrence in the surgical bed is common following resection alone for BM; therefore, several trials (41, 42) reported the potential benefit of postoperative radiotherapy with WBRT or RS. However, it should be noted that only a portion of the studies explicitly described whether patients in the surgical group received adjuvant radiotherapy.

### Prognostic Factors

From the pooled results, we observed that the presence of ECM may be associated with poor survival outcomes. A recent study (43) focusing on the relevance of extracranial metastatic patterns and the survival outcome of patients with CRC BM showed that patients with lung metastasis lived longer than those with liver metastasis, and concurrent liver and lung metastasis were associated with the worst survival outcome. However, only five studies (4, 12, 28, 30, 35) reported the survival outcome of patients with and without ECM, and the survival of patients with different metastatic patterns was not reported in the included studies.

The other significant factor predicting poor survival outcome was multiple BM. We believed that the presence of multiple metastases may affect the choice of local treatment for BM; however, the presence of multiple BM is not a contraindication for metastasectomy (44). Unfortunately, the criteria to determine surgery or radiotherapy for patients with CRC BM were not reported in studies included in our meta-analysis. In reality, the treatment choice for surgical resection or radiotherapy for CRC BM may not only be influenced by the number of metastases but also by the anatomic location, surgically accessibility, the condition of patients, and the experience of surgeons. The effect of the potential allocation bias could not be clearly clarified in our study and should be addressed in future studies.

### Comparison With the Previous Synthesis

In a recently published systematic review (45), Müller et al. included 86 articles to investigate the incidence, symptoms, diagnosis, treatment, and prognosis of CRC BM. Although it provided a comprehensive perspective regarding CRC BM, the inclusion criteria were not clearly defined in their method. In contrast, we emphasized the current evidence on local treatment modalities. As a result, we focused on studies comparing the treatment outcomes and included fewer studies than the aforementioned review. Although the authors summarized predictors for poor survival in CRC BM, such as advanced age, low KPS, ECM, multiple BM, and elevated carcinoembryonic antigen, direct comparison among therapeutic approaches was still omitted. Our meta-analysis is the first one focusing on comparative survival outcomes of patients with CRC BM receiving different local treatment modalities.

## Limitations

The present meta-analysis has limitations. First, there were several studies not included in our review due to insufficient data for survival outcomes based on different treatments. Second, all included studies were retrospective cohorts and the detailed baseline characteristics in each treatment group were limited; therefore, the bias due to confounders would not be well-adjusted. Third, the paucity of information on adjuvant radiotherapy in the surgical group hindered an accurate assessment of the treatment modalities. Fourth, our results supported the survival benefit of surgical resection compared with radiotherapy for BM; however, the selection criteria for a patient of surgery or radiotherapy were not detailed in most of the studies included in our meta-analysis.

Last, the lack of details regarding the chemotherapeutic agents in the included studies limited our assessment of their effect. Similarly, although Fountzilas et al. (31) reported survival benefits of patients receiving biologic agents after BM, no relevant data in other included studies were reported. Thus, it is difficult to value the effect of chemotherapeutic and biologic agents for patients with CRC BM treated with surgical resection or radiotherapy.

## Conclusions

This systematic review and meta-analysis involving patients with CRC BM have demonstrated the benefit of aggressive neurosurgical management in suitable patients. Additionally, identifying prognosticators including ECM and multiple BM would aid in the treatment and decision-making in these patients. Considering the potential limitations, further prospective studies are warranted.

## Data Availability Statement

The original contributions presented in the study are included in the article/Supplementary Material, further inquiries can be directed to the corresponding author.

## Author Contributions

YC and C-EW contributed to study conceptualization, data curation, statistical analysis, data interpretation, drafting of the manuscript, and has full access to all the data in the study. P-HL contributed to statistical analysis, data interpretation, and critical review of the manuscript. C-CH contributed to data interpretation and critical review of the manuscript. J-SL contributed to study conceptualization, data interpretation, critical review of the manuscript, and is responsible for the integrity and accuracy of data analysis. All authors contributed to the article and approved the submitted version.

## Conflict of Interest

The authors declare that the research was conducted in the absence of any commercial or financial relationships that could be construed as a potential conflict of interest.

## Publisher's Note

All claims expressed in this article are solely those of the authors and do not necessarily represent those of their affiliated organizations, or those of the publisher, the editors and the reviewers. Any product that may be evaluated in this article, or claim that may be made by its manufacturer, is not guaranteed or endorsed by the publisher.
