# Supplementary material for: Survival Outcome of Surgical Resection vs. Radiotherapy in Brain Metastasis From Colorectal Cancer: A Meta-Analysis
Source: Front Med (Lausanne). 2022 Mar 8;9:768896. doi: 10.3389/fmed.2022.768896 (PMC8957984; doi:10.3389/fmed.2022.768896)

**Supplemental File**

**Survival outcome of surgical resection versus radiotherapy in brain metastasis from colorectal cancer: A meta-analysis**

**eMethod 1.** Preferred Reporting Items for Systematic Reviews and Meta-Analyses (PRISMA) 2020 checklist

**eMethod 2.** Meta-analysis Of Observational Studies in Epidemiology (MOOSE) checklist

**eMethod 3.** Search strategy

**eMethod 4.** Data synthesis

**eTable 1**. Reasons for the exclusion of studies after the full-text inspection

**eTable 2**. Risk of bias assessment for overall survival: Risk Of Bias In Non-randomized Studies- of Intervention (ROBINS-I)

**eFigure 1**. Forest plot for overall survival in patients receiving surgical resection versus radiotherapy with subgroup analysis based on univariate and multivariate hazard ratios

**eFigure 2**. Forest plot for overall survival based on studies published year

**eFigure 3**. Forest plot for overall survival based on different patient number of the included studies **eFigure 4**. Forest plot for overall survival based on extracranial metastasis

**eFigure 5**. Forest plot for overall survival based on numbers of brain metastasis

**eFigure 6**. Forest plot for overall survival based on synchronous or metachronous brain metastasis

**eFigure 7**. Forest plot for overall survival based on location of primary tumor

**eFigure 8.** Assessment of publication bias of long-term survival through funnel plots

**eFigure 9.** Linear regression test of funnel plot asymmetry.

**eMethod 1.** PRISMA 2020 checklist

| **Section and Topic** | **Item #** | **Checklist item** | **Location where item is reported** |
| --- | --- | --- | --- |
| **TITLE** | | |  |
| Title | 1 | Identify the report as a systematic review. | Page 1 |
| **ABSTRACT** | | |  |
| Abstract | 2 | See the PRISMA 2020 for Abstracts checklist. | Page 3, 4 |
| **INTRODUCTION** | | |  |
| Rationale | 3 | Describe the rationale for the review in the context of existing knowledge. | Page 5, 6 |
| Objectives | 4 | Provide an explicit statement of the objective(s) or question(s) the review addresses. | Page 5, 6 |
| **METHODS** | | |  |
| Eligibility criteria | 5 | Specify the inclusion and exclusion criteria for the review and how studies were grouped for the syntheses. | Page 7 |
| Information sources | 6 | Specify all databases, registers, websites, organisations, reference lists and other sources searched or consulted to identify studies. Specify the date when each source was last searched or consulted. | Page 6 |
| Search strategy | 7 | Present the full search strategies for all databases, registers and websites, including any filters and limits used. | eMethod 3 |
| Selection process | 8 | Specify the methods used to decide whether a study met the inclusion criteria of the review, including how many reviewers screened each record and each report retrieved, whether they worked independently, and if applicable, details of automation tools used in the process. | Page 6 |
| Data collection process | 9 | Specify the methods used to collect data from reports, including how many reviewers collected data from each report, whether they worked independently, any processes for obtaining or confirming data from study investigators, and if applicable, details of automation tools used in the process. | Page 7 |
| Data items | 10a | List and define all outcomes for which data were sought. Specify whether all results that were compatible with each outcome domain in each study were sought (e.g. for all measures, time points, analyses), and if not, the methods used to decide which results to collect. | Page 7 |
|  | 10b | List and define all other variables for which data were sought (e.g. participant and intervention characteristics, funding sources). Describe any assumptions made about any missing or unclear information. | Page 7 |
| Study risk of bias assessment | 11 | Specify the methods used to assess risk of bias in the included studies, including details of the tool(s) used, how many reviewers assessed each study and whether they worked independently, and if applicable, details of automation tools used in the process. | Page 8 |
| Effect measures | 12 | Specify for each outcome the effect measure(s) (e.g. risk ratio, mean difference) used in the synthesis or presentation of results. | Page 8, 9 |
| Synthesis methods | 13a | Describe the processes used to decide which studies were eligible for each synthesis (e.g. tabulating the study intervention characteristics and comparing against the planned groups for each synthesis (item #5)). | Page 7 |
|  | 13b | Describe any methods required to prepare the data for presentation or synthesis, such as handling of missing summary statistics, or data conversions. | Page 8 |
|  | 13c | Describe any methods used to tabulate or visually display results of individual studies and syntheses. | eMethod 4 |
|  | 13d | Describe any methods used to synthesize results and provide a rationale for the choice(s). If meta-analysis was performed, describe the model(s), method(s) to identify the presence and extent of statistical heterogeneity, and software package(s) used. | eMethod 4 |
|  | 13e | Describe any methods used to explore possible causes of heterogeneity among study results (e.g. subgroup analysis, meta-regression). | Page 8, 9 |
|  | 13f | Describe any sensitivity analyses conducted to assess robustness of the synthesized results. | Page 8 |
| Reporting bias assessment | 14 | Describe any methods used to assess risk of bias due to missing results in a synthesis (arising from reporting biases). | Page 9 |
| Certainty assessment | 15 | Describe any methods used to assess certainty (or confidence) in the body of evidence for an outcome. | N/A |
| **RESULTS** | | |  |
| Study selection | 16a | Describe the results of the search and selection process, from the number of records identified in the search to the number of studies included in the review, ideally using a flow diagram. | Page 9, 10 |
|  | 16b | Cite studies that might appear to meet the inclusion criteria, but which were excluded, and explain why they were excluded. | eTable 1 |
| Study characteristics | 17 | Cite each included study and present its characteristics. | Page 9, 10 |
| Risk of bias in studies | 18 | Present assessments of risk of bias for each included study. | Page 10 |
| Results of individual studies | 19 | For all outcomes, present, for each study: (a) summary statistics for each group (where appropriate) and (b) an effect estimate and its precision (e.g. confidence/credible interval), ideally using structured tables or plots. | Figure 2 |
| Results of syntheses | 20a | For each synthesis, briefly summarise the characteristics and risk of bias among contributing studies. | eTable 2 |
|  | 20b | Present results of all statistical syntheses conducted. If meta-analysis was done, present for each the summary estimate and its precision (e.g. confidence/credible interval) and measures of statistical heterogeneity. If comparing groups, describe the direction of the effect. | Page 10, 11 |
|  | 20c | Present results of all investigations of possible causes of heterogeneity among study results. | Page 10, 11 |
|  | 20d | Present results of all sensitivity analyses conducted to assess the robustness of the synthesized results. | Page 10, 11 |
| Reporting biases | 21 | Present assessments of risk of bias due to missing results (arising from reporting biases) for each synthesis assessed. | Page 11 |
| Certainty of evidence | 22 | Present assessments of certainty (or confidence) in the body of evidence for each outcome assessed. | N/A |
| **DISCUSSION** | | |  |
| Discussion | 23a | Provide a general interpretation of the results in the context of other evidence. | Page 12 |
|  | 23b | Discuss any limitations of the evidence included in the review. | Page 16 |
|  | 23c | Discuss any limitations of the review processes used. | Page 16 |
|  | 23d | Discuss implications of the results for practice, policy, and future research. | Page 16 |
| **OTHER INFORMATION** | | |  |
| Registration and protocol | 24a | Provide registration information for the review, including register name and registration number, or state that the review was not registered. | Page 6 |
|  | 24b | Indicate where the review protocol can be accessed, or state that a protocol was not prepared. | Page 6 |
|  | 24c | Describe and explain any amendments to information provided at registration or in the protocol. | Page 6 |
| Support | 25 | Describe sources of financial or non-financial support for the review, and the role of the funders or sponsors in the review. | N/A |
| Competing interests | 26 | Declare any competing interests of review authors. | N/A |
| Availability of data, code and other materials | 27 | Report which of the following are publicly available and where they can be found: template data collection forms; data extracted from included studies; data used for all analyses; analytic code; any other materials used in the review. | N/A |

**eMethod 2.** MOOSE checklist

| Reporting Criteria | Reported (Yes/No) | Reported on Page No. | Brief description |
| --- | --- | --- | --- |
| Reporting of Background |  |  |  |
| Problem definition | Yes | 5, 6 | Given the incongruent literature, the optimal therapeutic approach for brain metastasis from colorectal cancer remains to be investigated. |
| Hypothesis statement | Yes | 5, 6 | Surgical resection may bring survival benefit |
| Description of Study Outcome(s) | Yes | 7 | Overall survival after brain metastasis |
| Type of exposure or intervention used | Yes | 7 | Surgical resection or radiotherapy |
| Type of study design used | Yes | 7 | Prospective/retrospective cohort studies |
| Study population | Yes | 7 | Adults with brain metastasis from colorectal cancer |
| Reporting of Search Strategy |  |  |  |
| Qualifications of searchers (eg, librarians and investigators) | Yes | 6 | Y.C and C.E.W |
| Search strategy, including time period included in the synthesis and keywords | Yes | eMethod 3 | See eMethod 3 |
| Effort to include all available studies, including contact with authors | Yes | eMethod 3 | We searched bibliographies of retrieved references.  It is unnecessary to contact authors as the data were publicly available. |
| Databases and registries searched | Yes | eMethod 3 | Embase, Medline and the Cochrane library |
| Search software used, name and version, including special features used (eg, explosion) | Yes | eMethod 3 | Endnote X 9.3 was used to manage reference |
| Use of hand searching (eg, reference lists of obtained articles) | Yes | eMethod 3 | We searched bibliographies of retrieved references. |
| List of citations located and those excluded, including justification | Yes | Figure 1 | Details of the literature search process are outlined in the PRISMA flow diagram. The citation list for excluded studies is available upon request. |
| Method for addressing articles published in languages other than English | N/A | 7 | We only included articles published in English |
| Method of handling abstracts and unpublished studies | Yes | 7 | We did not include any conference abstract. |
| Description of any contact with authors | Yes | eMethod 3 | It is unnecessary to contact authors as the data were publicly available. |
| Reporting of Methods |  |  |  |
| Description of relevance or appropriateness of studies assembled for assessing the hypothesis to be tested | Yes | 7 | 1. Studies of adults with CRC BM were included 2. Studies reporting comparative survival outcome of radiotherapy or surgical resection and using overall survival (OS) as endpoint were included. |
| Rationale for the selection and coding of data (eg, sound clinical principles or convenience) | Yes | 7 | Two investigators (Y.C and C.E.W) independently extracted relevant information from eligible articles. |
| Documentation of how data were classified and coded (eg, multiple raters, blinding, and interrater reliability) | Yes | 7 |  |
| Assessment of confounding (eg, comparability of cases and controls in studies where appropriate | Yes | 7 | Two reviewers (Y.C and C.E.W) independently completed a critical appraisal of included literature by using the Risk Of Bias In Non-randomized Studies - of Interventions (ROBINS-I) tool. |
| Reporting Criteria |  |  |  |
| Assessment of study quality, including blinding of quality assessors; stratification or regression on possible predictors of study results | Yes | 8 | ROBINS-I tool was used for assessment of study quality. |
| Assessment of heterogeneity | Yes | 8 | Heterogeneity was assessed using I^2^ statistics proposed by Higgins and Thompson. |
| Description of statistical methods (eg, complete description of fixed or random effects models, justification of whether the chosen models account for predictors of study results, dose-response models, or cumulative meta-analysis) in sufficient detail to be replicated | Yes | 8, eMthod 4 | We performed meta-analysis using RStudio’s ‘‘metafor’’ package was used for all analyses. |
| Provision of appropriate tables and graphics | Yes | Figure and Table | We included tables for illustrating details of included the studies and figures demonstrating a flow chart of study identification and the results of the meta-analyses. |
| Reporting of Results |  |  |  |
| Table giving descriptive information for each study included | Yes | Table 1 | Details are in Table 1 |
| Results of sensitivity testing (eg, subgroup analysis) | Yes | 10, 11 | Subgroup analysis was performed based on radiotherapy type and crude or adjusted hazard ratio |
| Indication of statistical uncertainty of findings | Yes | 10, 11 | 95% confidence intervals and I^2^ values were presented with all effect estimates |
| Reporting of Discussion |  |  |  |
| Quantitative assessment of bias (eg, publication bias) | Yes | 11 | The visually symmetrical funnel plot with Egger’s test result indicated no potential publication bias. |
| Justification for exclusion (eg, exclusion of non–English-language citations) | Yes | 7 | Studies were excluded based on the pre-specified eligibility criteria in Method. |
| Assessment of quality of included studies | Yes | eTable 2 | See eTable 2 |
| Reporting of Conclusions |  |  |  |
| Consideration of alternative explanations for observed results | Yes | 14 | Although the survival benefit of surgical resection for BM was observed in our analysis, this beneficial effect may be potentially associated with the adjuvant radiotherapy administered following surgery. |
| Generalization of the conclusions (ie, appropriate for the data presented and within the domain of the literature review) | Yes | 16 | Our study demonstrated the benefit of aggressive neurosurgical management in suitable patients. |
| Guidelines for future research | Yes | 16 | We propose that prospective large multicenter studies should be undertaken to overcome the limitations. |
| Disclosure of funding source | N/A |  |  |

**eMethod 3.** Search strategy

**Embase**

| Search Number | Search Description | Numbers of results |
| --- | --- | --- |
| 1 | (brain or cerebral or cerebrum or cerebellar or cerebellum or hemisphere or supratentorial or infratentorial or intracranial):ti,ab,kw,de | 2627699 |
| 2 | 'brain'/exp | 1535257 |
| 3 | ((metastasis or metastatic)):ti,ab,kw,de | 922547 |
| 4 | 'metastasis '/exp | 704913 |
| 5 | ((colon or colorectal or rectum or cecum)):ti,ab,kw,de | 754704 |
| 6 | (cancer or carcinoma or malignant tumor or malignancy or neoplasm):ti,ab,kw,de | 2746574 |
| 7 | 'colorectal carcinoma'/exp | 25941 |
| 8 | (1 OR 2) AND (3 OR 4 ) AND ((5 AND 6) OR 7) and [embase]/lim | 3894 |

**Medline**

| Search Number | Search Description | Numbers of results |
| --- | --- | --- |
| 1 | (brain or cerebral or cerebrum or cerebellar or cerebellum or hemisphere or supratentorial or infratentorial or intracranial).mp | 1875095 |
| 2 | exp "Brain" | 1250170 |
| 3 | ((metastasis or metastatic)).mp | 527723 |
| 4 | exp "Neoplasm Metastasis" | 211602 |
| 5 | (colon or colorectal or rectum or cecum).mp | 406465 |
| 6 | (cancer or carcinoma or malignant tumor or malignancy or neoplasm).mp | 2728453 |
| 7 | exp "Colorectal Neoplasm" | 212670 |
| 8 | (1 OR 2) AND (3 OR 4) AND ((5 AND 6) OR 7) | 1290 |
| [mp=title, abstract, original title, name of substance word, subject heading word, floating sub-heading word, keyword heading word, organism supplementary concept word, protocol supplementary concept word, rare disease supplementary concept word, unique identifier, synonyms] | | |

**Cochrane library**

| Search Number | Search Description | Numbers of results |
| --- | --- | --- |
| 1 | (brain or cerebral or cerebrum or cerebellar or cerebellum or hemisphere or supratentorial or infratentorial or intracranial): ti,ab,kw | 91944 |
| 2 | MeSH descriptor: [Brain] explode all trees | 12003 |
| 3 | (metastasis or metastatic):ti,ab,kw | 41837 |
| 4 | MeSH descriptor: [ Neoplasm Metastasis] explode all trees | 5298 |
| 5 | (colon or colorectal or rectum or cecum): ti,ab,kw | 36676 |
| 6 | (cancer or carcinoma or malignant tumor or malignancy or neoplasm): ti,ab,kw | 210786 |
| 7 | MeSH descriptor: [ Colorectal Neoplasm] explode all trees | 8605 |
| 8 | (1 OR 2) AND (3 OR 4) AND (5 AND 6 OR 7) in Trials | 77 |

It is unnecessary to contact authors as the data were publicly available.

**eMethod 4.** Data synthesis

We used Rstudio with metafor packages to conduct statistical analysis**【metafor】package**
**Random-effects model**
**Generic inverse variance meta-analysis (metagen)**

**DerSimonian and Laird method as a heterogeneity estimator**

**Several plots for meta-analysis: Forest plot (forest)**

**Publication bias and Egger’s test: (metabias)**

**Overall survival**

metaresult=metagen(logHR, selogHR, studlab=year,sm="HR",data=OS, method.tau="DL",comb.random=TRUE,comb.fixed=FALSE)

***Subgroup analysis***

metaresult=metagen(logHR, selogHR, studlab=year,sm="HR",data=OS,method.tau="DL",comb.random=TRUE,comb.fixed=FALSE, byvar=RT)

metaresult=metagen(logHR, selogHR, studlab=year,sm="HR",data=OS,method.tau="DL",comb.random=TRUE,comb.fixed=FALSE, byvar=HR)

metaresult=metagen(logHR, selogHR, studlab=year,sm="HR",data=OS,method.tau="DL",comb.random=TRUE,comb.fixed=FALSE, byvar=Publication)

metaresult=metagen(logHR, selogHR, studlab=year,sm="HR",data=OS,method.tau="DL",comb.random=TRUE,comb.fixed=FALSE, byvar=Patients)

***Forest plot***

forest (metaresult, layout="RevMan5", lab.e="OP", lab.c="RT", xlab="Favors surgical resection Fvors radiotherapy", ff.xlab="bold", col.by="black", pooled.events=F, comb.random=T, comb.fixed=F, col.diamond.random=("red"), col.diamond.lines.random="red", col.square = 'blue', col.square.lines = 'blue', test.subgroup.random=TRUE)

***Funnel plot and Egger’s test***

funnel (metaresult, comb.random = F, contour.levels = c(0.9,0.95,0.99), col.contour = c("dark blue","blue","light blue"), ref = exp(metaresult.or$TE.fixed))

metaresult.bias <- metabias(metaresult,method.bias="linreg",plotit=T)

**eTable 1. Reasons for the exclusion of studies after the full-text inspection**

**Insufficient data for comparison of overall survival**

1. Navarria P, Minniti G, Clerici E, et al. Brain metastases from primary colorectal cancer: is radiosurgery an effective treatment approach? Results of a multicenter study of the radiation and clinical oncology Italian association (AIRO). *The British journal of radiology.* 2020;93(1116):20200951.

2. Quan J, Ma C, Sun P, et al. Brain metastasis from colorectal cancer: clinical characteristics, timing, survival and prognostic factors. *Scandinavian Journal of Gastroenterology.* 2019;54(11):1370-1375.

3. Kim D-Y, Ryu C-G, Jung E-J, Paik J-H, Hwang D-Y. Brain metastasis from colorectal cancer: a single center experience. *Annals of surgical treatment and research.* 2018;94(1):13-18.

4. Kim BH, Park HJ, Kim K, et al. Novel graded prognostic assessment for colorectal cancer patients with brain metastases. *International journal of clinical oncology.* 2018;23(6):1112-1120.

5. Mongan JP, Fadul CE, Cole BF, et al. Brain metastases from colorectal cancer: Risk factors, incidence, and the possible role of chemokines. *Clinical Colorectal Cancer.* 2009;8(2):100-105.

6. D'Andrea G, Isidori A, Caroli E, Orlando ER, Salvati M. Single cerebral metastasis from colorectal adenocarcinoma. *Neurosurgical Review.* 2004;27(1):55-57.

7. Schoeggl A, Kitz K, Reddy M, Zauner C. Stereotactic radiosurgery for brain metastases from colorectal cancer. *International journal of colorectal disease.* 2002;17(3):150-155.

8. Wroński M, Arbit E. Resection of brain metastases from colorectal carcinoma in 73 patients. *Cancer.* 1999;85(8):1677-1685.

9. Ko FC, Liu JM, Chen WS, Chiang JK, Lin TC, Lin JK. Risk and patterns of brain metastases in colorectal cancer: 27-year experience. *Diseases of the colon and rectum.* 1999;42(11):1467-1471.

10. Farnell GF, Buckner JC, Cascino TL, O'Connell MJ, Schomberg PJ, Suman V. Brain metastases from colorectal carcinoma: The long term survivors. *Cancer.* 1996;78(4):711-716.

11. Cascino TL, Leavengood JM, Kemeny N, Posner JB. Brain metastases from colon cancer. *Journal of neuro-oncology.* 1983;1(3):203-209.

12. Zorrilla M, Alonso V, Herrero A, et al. Brain metastases from colorectal carcinoma. *Tumori.* 2001;87(5):332-334.

13. Naito H, Sasaki M, Kondo K, et al. Radical treatment of brain metastasis of colorectal-cancer. *Oncology reports.* 1994;1(6):1203-1205.

**Not reporting survival outcome of surgical resection versus radiotherapy**

14. Thurmaier J, Heinemann V, Engel J, et al. Patients with colorectal cancer and brain metastasis: The relevance of extracranial metastatic patterns predicting time intervals to first occurrence of intracranial metastasis and survival. *International journal of cancer.* 2021;148(8):1919-1927.

15. Shindorf ML, Jafferji MS, Goff SL. Incidence of Asymptomatic Brain Metastases in Metastatic Colorectal Cancer. *Clinical colorectal cancer.* 2020;19(4):263-269.

16. Scripcariu V, Ciobanu Apostol DG, Dumitrescu GF, Turliuc MD, Sava A. Clinical, histopathological and immunohistochemical features of brain metastases originating in colorectal cancer: a series of 27 consecutive cases. *Romanian journal of morphology and embryology = Revue roumaine de morphologie et embryologie.* 2020;61(1):81-93.

17. Quan J-C, Guan X, Ma C-X, et al. Prognostic scoring system for synchronous brain metastasis at diagnosis of colorectal cancer: A population-based study. *World journal of gastrointestinal oncology.* 2020;12(2):195-204.

18. Lei S, Ge Y, Tian S, et al. Colorectal Cancer Metastases to Brain or Bone and the Relationship to Primary Tumor Location: a Population-Based Study. *Journal of gastrointestinal surgery : official journal of the Society for Surgery of the Alimentary Tract.* 2020;24(8):1833-1842.

19. Imaizumi J, Shida D, Narita Y, et al. Prognostic factors of brain metastases from colorectal cancer. *BMC cancer.* 2019;19(1):755.

20. Chahine G, Ibrahim T, Felefly T, et al. Colorectal cancer and brain metastases: An aggressive disease with a different response to treatment. *Tumori.* 2019;105(5):427-433.

21. Yang L, He W, Xie Q, et al. Brain metastases in newly diagnosed colorectal cancer: A population-based study. *Cancer Management and Research.* 2018;10:5649-5658.

22. Nozawa H, Ishihara S, Kawai K, et al. Brain Metastasis from Colorectal Cancer: Predictors and Treatment Outcomes. *Oncology.* 2017;93(5):309-314.

23. Christensen TD, Palshof JA, Larsen FO, et al. Risk factors for brain metastases in patients with metastatic colorectal cancer. *Acta oncologica (Stockholm, Sweden).* 2017;56(5):639-645.

24. Tevlin R, Larkin JO, Hyland JM, O'Connell PR, Winter DC. Brain metastasis from colorectal carcinoma: a single cancer centre experience. *Irish journal of medical science.* 2015;184(3):673-675.

25. Michl M, Thurmaier J, Schubert-Fritschle G, et al. Brain Metastasis in Colorectal Cancer Patients: Survival and Analysis of Prognostic Factors. *Clinical Colorectal Cancer.* 2015;14(4):281-290.

26. Skeie BS, Enger PØ, Ganz JC, et al. Gamma knife surgery of colorectal brain metastases: A high prescription dose of 25 Gy may improve growth control. *World Neurosurgery.* 2013;79(3-4):525-536.

27. Tan W-S, Ho K-S, Eu K-W. Brain metastases in colorectal cancers. *World journal of surgery.* 2009;33(4):817-821.

28. Tokoro T, Okuno K, Hida J-c, et al. Prognostic factors for patients with advanced colorectal cancer and symptomatic brain metastases. *Clinical colorectal cancer.* 2014;13(4):226-231.

29. Roussille P, Auvray M, Vansteene D, et al. Prognostic factors of colorectal cancer patients with brain metastases. *Radiotherapy and oncology : journal of the European Society for Therapeutic Radiology and Oncology.* 2021;158:67-73.

30. Nieder C, Hintz M, Grosu AL. Colorectal cancer metastatic to the brain: analysis of prognostic factors and impact of KRAS mutations on presentation and outcome. *Clinical & translational oncology : official publication of the Federation of Spanish Oncology Societies and of the National Cancer Institute of Mexico.* 2016;18(1):88-92.

31. Aprile G, Zanon E, Tuniz F, et al. Neurosurgical management and postoperative whole-brain radiotherapy for colorectal cancer patients with symptomatic brain metastases. J Cancer Res Clin Oncol. 2009;135(3):451–457.

32. Fowler A, Cook R, Biggs M, et al. Survival of patients following neurosurgical treatment of colorectal adenocarcinoma metastasis in the Northern Sydney-Central Coast area. J Clin Neurosci. 2008;15(9):998–1004.

33. Gu XD, Cai YT, Zhou YM, et al. Prognostic factors and multidisciplinary treatment modalities for brain metastases from colorectal cancer: analysis of 93 patients. BMC Cancer 2015;15:902.

34. Mege D, Ouaissi M, Fuks D, et al. Patients with brain metastases from colorectal cancer are not condemned. Anticancer Res. 2013;33(12):5645–5648.

35. Jiang XB, Yang QY, Sai K, Zhang XH, Chen ZP, Mou YG. Brain metastases from colorectal carcinoma: a description of 60 cases in a single Chinese cancer center. *Tumor Biology.* 2011:1-8.

36. Nieder C, Pawinski A, Balteskard L. Colorectal cancer metastatic to the brain: time trends in presentation and outcome. *Oncology.* 2009;76(5):369-374.

37. Onodera H, Nagayama S, Tachibana T, Fujimoto A, Imamura M. Brain metastasis from colorectal cancer. *International Journal of Colorectal Disease.* 2005;20(1):57-61.

**Single treatment modality**

38. Rades D, Hansen HC, Janssen S, Schild SE. Diagnosis-specific WBRT-30-CRC Score for Estimating Survival of Patients Irradiated for Brain Metastases from Colorectal Cancer. *Anticancer research.* 2019;39(5):2569-2574.

39. Paix A, Antoni D, Adeduntan R, Noel G. Stereotactic radiation therapy of brain metastases from colorectal cancer: A single institution cohort. *Cancer radiotherapie : journal de la Societe francaise de radiotherapie oncologique.* 2017;21(3):199-204.

40. Matsunaga S, Shuto T, Kawahara N, Suenaga J, Inomori S, Fujino H. Gamma Knife surgery for brain metastases from colorectal cancer. *Journal of Neurosurgery.* 2011;114(3):782-789.

41. Amichetti M, Lay G, Dessi M, et al. Results of whole brain radiation therapy in patients with brain metastases from colorectal carcinoma. *Tumori.* 2005;91(2):163-167.

**Not reporting brain metastasis**

42. Shi T, Huang M, Han D, et al. Chemotherapy is associated with increased survival from colorectal signet ring cell carcinoma with distant metastasis: A Surveillance, Epidemiology, and End Results database analysis. *Cancer Medicine.* 2019;8(4):1930-1940.

**eTable 2**. Risk of bias assessment for overall survival: Risk Of Bias In Non-randomized Studies- of Intervention (ROBINS-I)


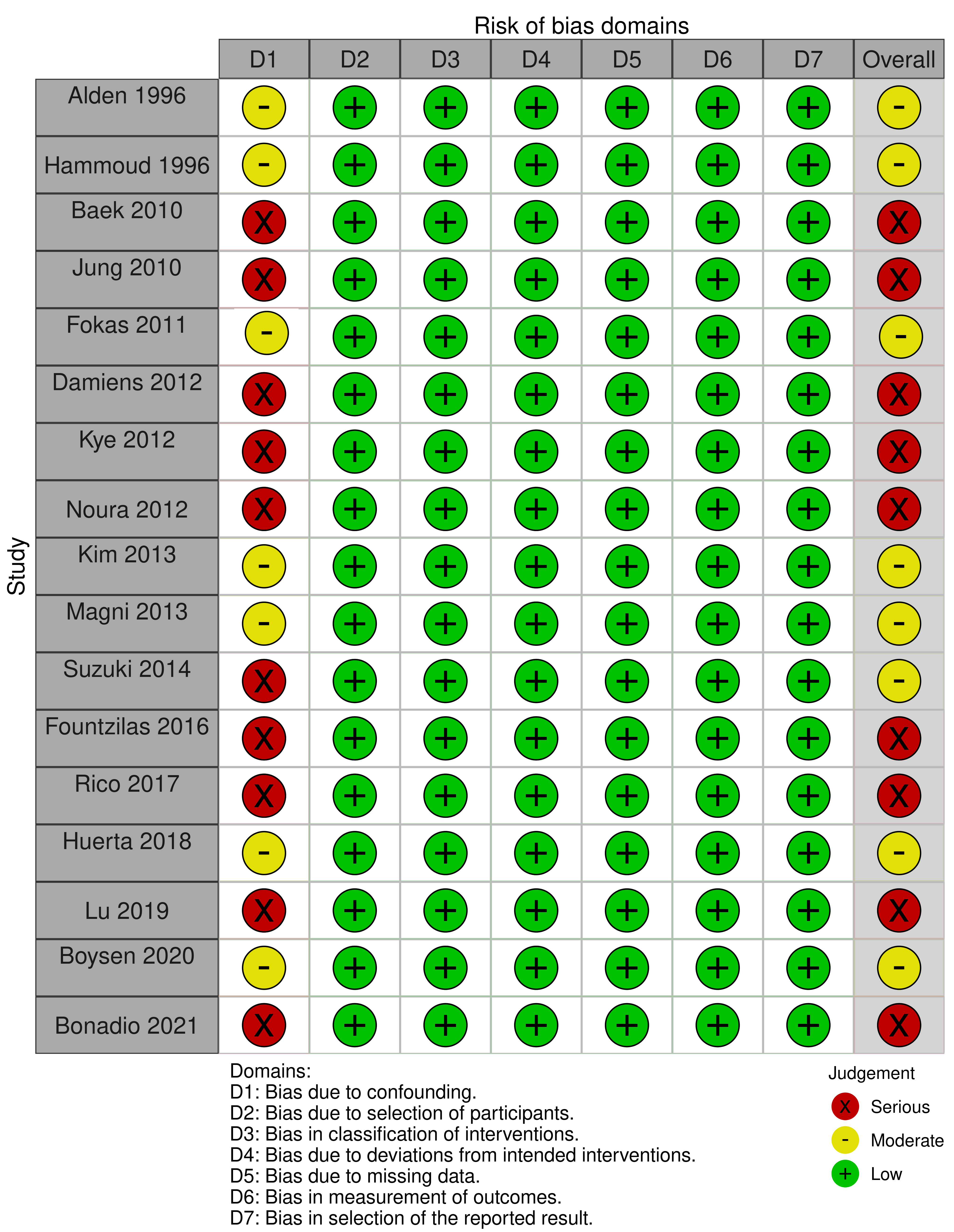


**eFigure 1**. Forest plot for overall survival in patients receiving surgical resection versus radiotherapy with subgroup analysis based on univariate and multivariate hazard ratios**.** Pooled HR with 95% CI was calculated under random-effects model. CI, confidence interval, CT, chemotherapy, HR, hazard ratio, RT, radiotherapy.

*surgical resection alone, **surgical resection ± RT, ***surgical resection + RT


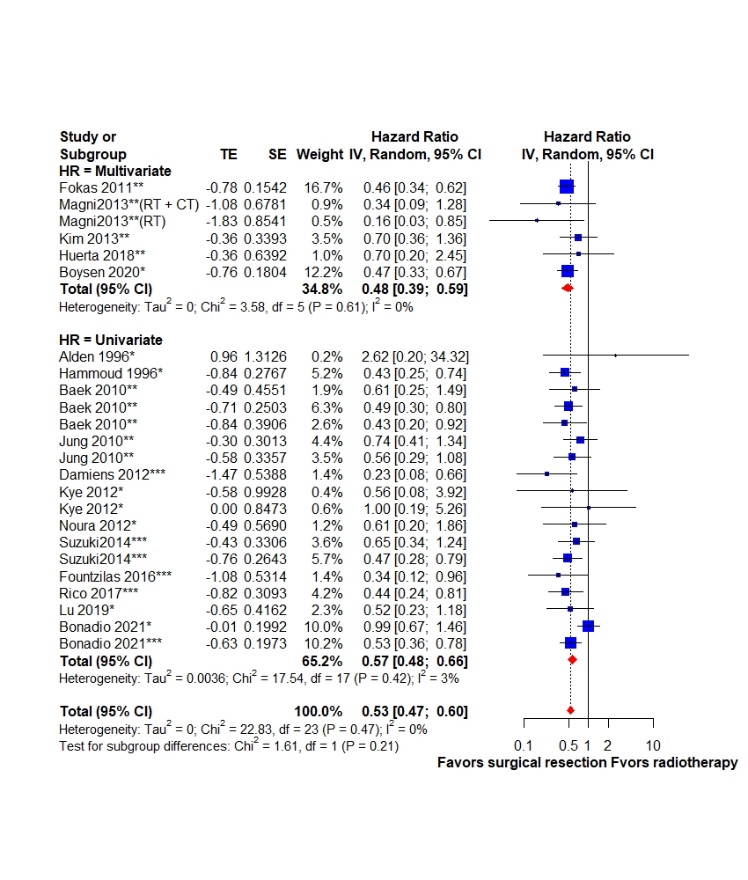


**eFigure 2**. Forest plot for overall survival in patients receiving surgical resection versus radiotherapy with subgroup analysis based on studies published within 5 years (after 2016) or more than 5 years ago (before 2016)**.** Pooled HR with 95% CI was calculated under random-effects model. CI, confidence interval, CT, chemotherapy, HR, hazard ratio, RT, radiotherapy. *surgical resection alone, **surgical resection ± RT, ***surgical resection + RT


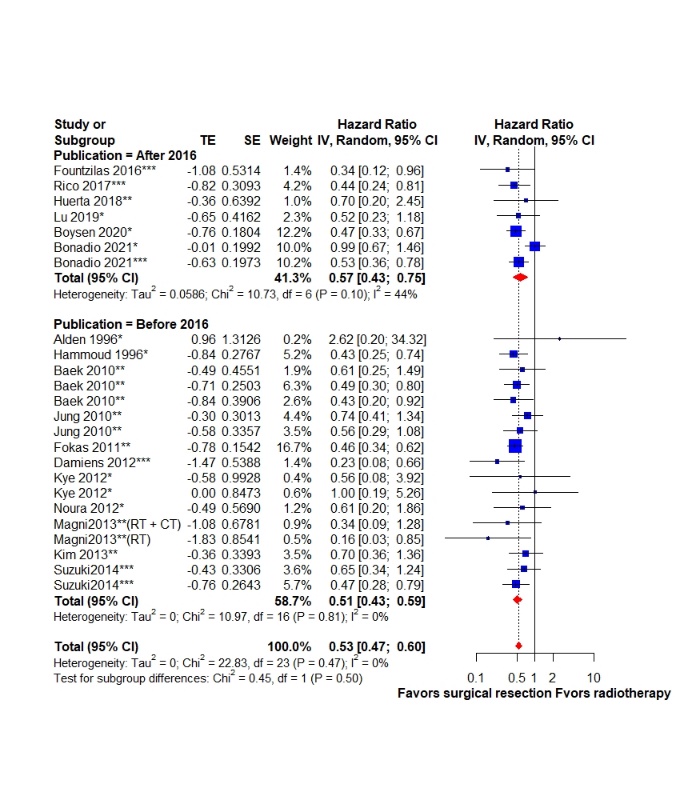


**eFigure 3**. Forest plot for overall survival in patients receiving surgical resection versus radiotherapy with subgroup analysis based on different patient number of the included studies**.** Pooled HR with 95% CI was calculated under random-effects model. CI, confidence interval, CT, chemotherapy, HR, hazard ratio, RT, radiotherapy.

*surgical resection alone, **surgical resection ± RT, ***surgical resection + RT


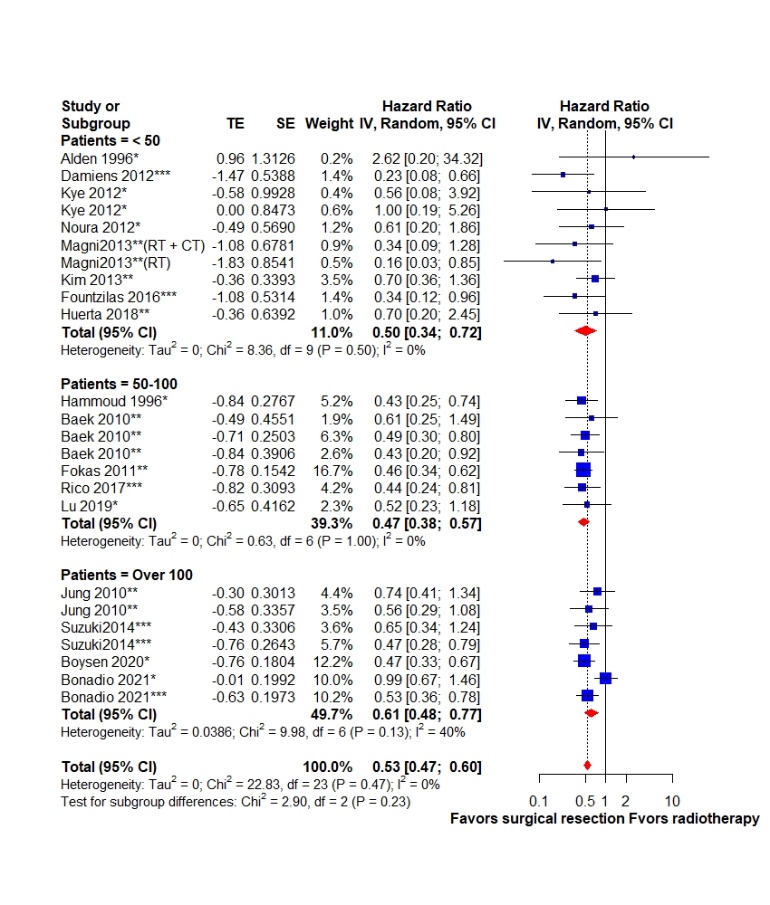


**eFigure 4**. Forest plot for overall survival based on extracranial metastasis

Pooled HR with 95% CI was calculated under random-effects model. CI, confidence interval, ECM, extracranial metastasis, HR, hazard ratio


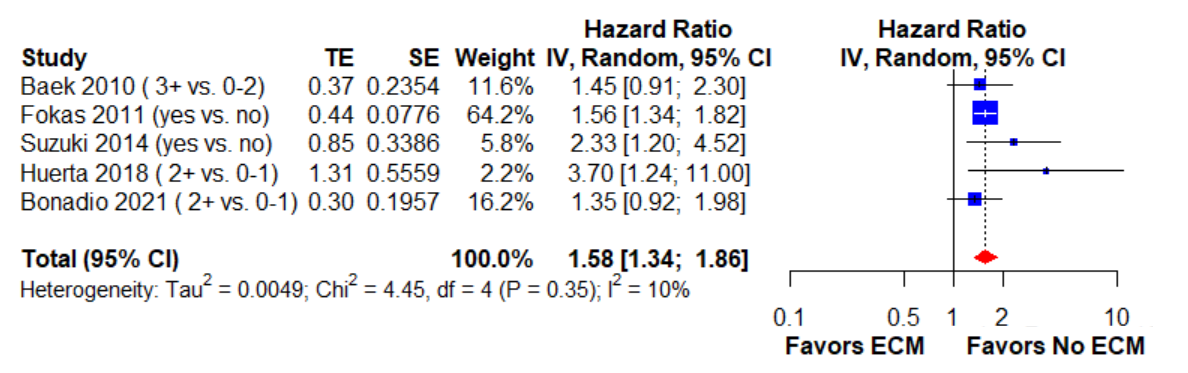


**eFigure 5**. Forest plot for overall survival based on numbers of brain metastasis

Pooled HR with 95% CI was calculated under random-effects model. BM, brain metastasis, CI, confidence interval, HR, hazard ratio

**
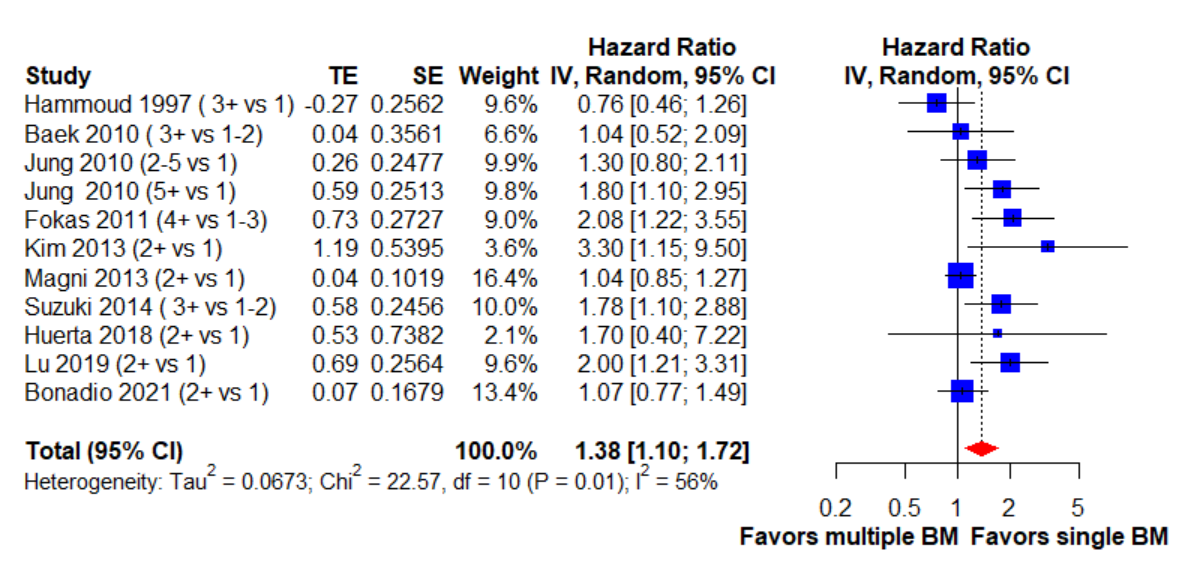
**

**eFigure 6**. Forest plot for overall survival based on synchronous or metachronous brain metastasis. Pooled HR with 95% CI was calculated under random-effects model. CI, confidence interval, HR, hazard ratio


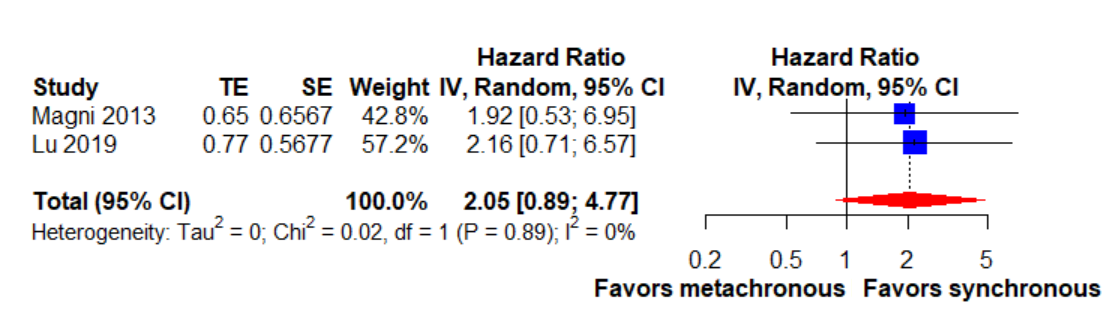


**eFigure 7**. Forest plot for overall survival based on location of primary tumor. Pooled HR with 95% CI was calculated under random-effects model. CI, confidence interval, HR, hazard ratio


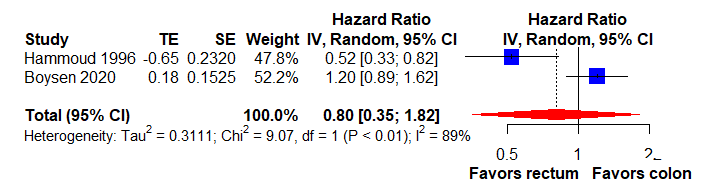


**eFigure 8.** Assessment of publication bias of long-term survival through funnel plots. Funnel plot demonstrates no significant asymmetry through visualization.


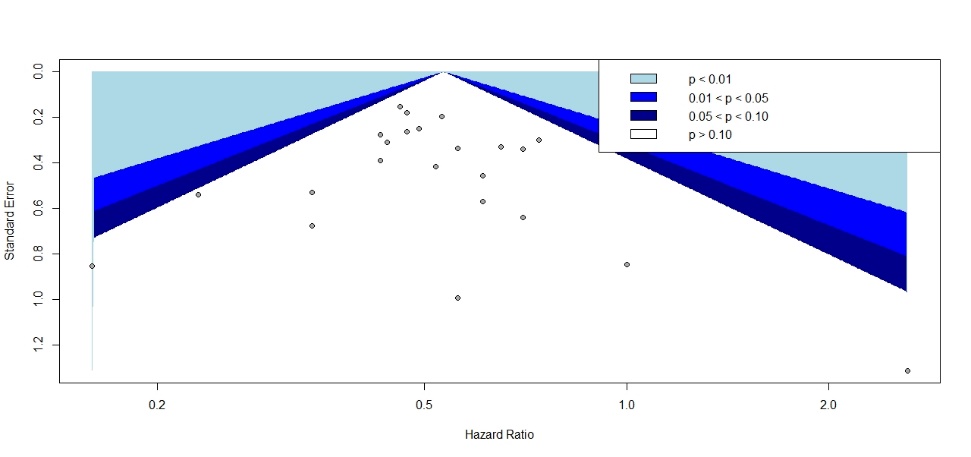


**eFigure 9.** Egger's test of funnel plot asymmetry. The result of Egger's test demonstrated no significant asymmetry. (P=0.85)


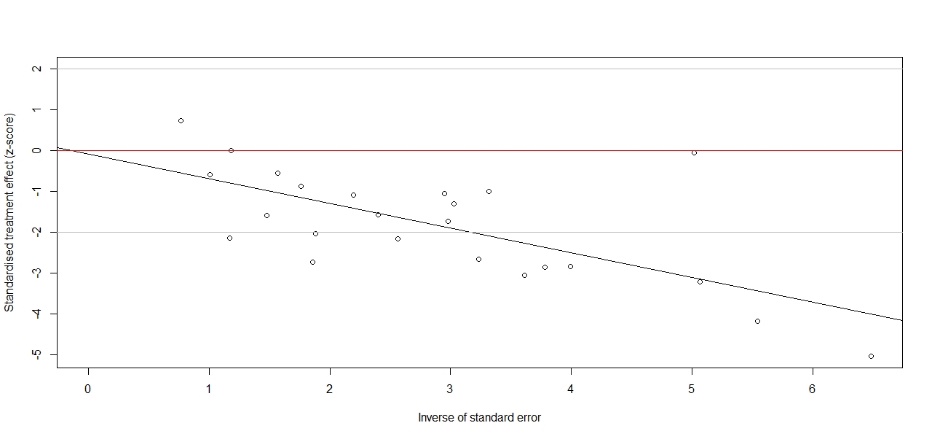

Supplement: Supplementary file 1 [file Data_Sheet_1.DOCX]
